# Association of liver enzyme levels and alveolar bone loss: A cross-sectional clinical study in Sado Island

**DOI:** 10.4317/jced.54555

**Published:** 2018-02-01

**Authors:** Ayumi Kuroki, Noriko Sugita, Shigeki Komatsu, Akio Yokoseki, Akihiro Yoshihara, Tetsuo Kobayashi, Kazutoshi Nakamura, Takeshi Momotsu, Naoto Endo, Kenji Sato, Ichiei Narita, Hiromasa Yoshie

**Affiliations:** 1 DDS, Division of Periodontology, Department of Oral Biological Science, Niigata University Graduate School of Medical and Dental Sciences, Niigata, Japan; 2 Assistant Professor, DDS, PhD, Division of Periodontology, Department of Oral Biological Science, Niigata University Graduate School of Medical and Dental Sciences, Niigata, Japan; 3 DDS, PhD, Sado General Hospital, Niigata, Japan; 4 Specially appointed Associate Professor, MD, PhD, Department of Molecular Neuroscience, Resource Branch for Brain Disease Research, Brain Research Institute, Niigata, Japan; 5 Professor, DDS, PhD, Department of Oral Health and Welfare, Niigata University Graduate School of Medical and Dental Sciences, Niigata, Japan; 6 Associate Professor, DDS, PhD, General Dentistry and Clinical Education Unit, Niigata University Medical and Dental Hospital, Niigata, Japan; 7 Professor, MD, PhD, Division of Preventive Medicine, Niigata University Graduate School of Medical and Dental Sciences, Niigata, Japan; 8 MD, PhD, Sado General Hospital, Niigata, Japan; 9 Professor, MD, PhD, Division of Orthopedic Surgery, Department of Regenerative and Transplant Medicine, Niigata University Graduate School of Medical and Dental Science, Niigata, Japan; 10 Professor, MD, PhD, Division of Clinical Nephrology and Rheumatology, Niigata University Graduate School of Medical and Dental Sciences, Niigata, Japan; 11 Professor, DDS, PhD, Division of Periodontology, Department of Oral Biological Science, Niigata University Graduate School of Medical and Dental Sciences, Niigata, Japan

## Abstract

**Background:**

The interaction of periodontopathic bacteria with host immune system induces the production of inflammatory mediators which leads to alveolar bone loss (ABL), the essential feature of periodontitis. Concurrently, periodontal diseases cause the elevation of blood cytokine levels, the alteration of gut microbiota and the dissemination of enterobacteria to the liver. Owing to these mechanisms, periodontal disease might be a risk for liver dysfunction. Several epidemiological studies have reported associations between periodontal diseases and liver dysfunction, although the association between ABL and liver dysfunction has not been investigated. This cross-sectional study determined if elevated serum liver enzyme levels were associated with ABL in Japanese adults.

**Material and Methods:**

Japanese adults living on Sado Island who visited Sado General Hospital were invited to participate in the study. Participants over 40 years of age who underwent dental panoramic radiography and blood tests were included. Drinking and smoking habits were self-administered. After excluding patients with edentulous jaw, diagnosed liver diseases, and those on dialysis, data from 44 men and 66 women with a mean age of 73 years were analyzed. The average percentage of ABL for each participant was calculated for mesial and distal sites of all remaining teeth. The levels of serum aspartate aminotransferase (AST), alanine aminotransferase (ALT) and gamma-glutamyltransferase (GGT) were determined. Univariate analyses were performed to select covariates to be put in multivariate analyses. The association between elevated serum liver enzyme levels and the highest quartile of ABL were assessed by multiple logistic regression analysis.

**Results:**

After adjusting for covariates, no significant association was found between elevated serum AST, ALT, or GGT levels as dependent variables and the highest quartile of ABL as an explanatory variable.

**Conclusions:**

There was no significant association between the elevation of serum liver enzyme levels and ABL in Japanese adults.

** Key words:**Liver enzymes, dental panoramic radiography, alveolar bone loss, Japanese adults.

## Introduction

Periodontal diseases are chronic inflammatory diseases in which periodontal tissues are destroyed by Gram-negative anaerobic bacteria such as Porphyromonas gingivalis (1). Periodontal pathogens are released from the sulcus into the bloodstream, and lead to transient bacteremia (2). The virulence factors of periodontal bacteria, including lipopolysaccharide (LPS), antigens, and exotoxins, interact with the host immune system inducing the production of inflammatory mediators, tumor necrosis factor-α (TNF-α), interleukin-1β (IL-1β), interleukin-6 (IL-6), receptor activator of nuclear factor kappa-B ligand (RANKL), and prostaglandin E2 (3-5). Owing to these mechanisms, periodontal diseases are thought to be associated with several systemic diseases including atherosclerosis, cardiovascular diseases, hyperlipidemia and rheumatoid arthritis (6-9).

In animal studies, periodontitis induced by nylon ligature or oral administration of LPS in rats caused decreased pericytes and liver steatosis (10,11). Periodontitis-induced rats receiving tooth brushing showed reduced serum LPS concentration and suppressed hepatic inflammation, steatosis and 8-hydroxydeoxyguanosin levels (12). In other reports, oral administration of *P. gingivalis* in mice induced altered gut microbiota and dissemination of enterobacteria to the liver as well as inflammatory changes in various tissues and organs (13,14). A cohort study in Japan suggested that infection with high-virulence *P. gingivalis* might be a risk factor for the contraction/progression of non-alcoholic fatty liver disease (15). The presence of periodontal pockets was associated with serum levels of gamma-glutamyltransferase (GGT) in Japanese adults independent of alcohol ingestion (16). Periodontal condition was associated with alanine aminotransferase (ALT) levels in Japanese men (17,18). Combined, these reports have shown the associations of periodontal diseases with hepatic metabolism.

Few reports have evaluated alveolar bone loss (ABL) as an indicator of periodontal diseases. Therefore, the present study assessed the association between the elevation of serum liver enzymes and ABL in Japanese adults living on Sado Island.

## Material and Methods

-Participants

**Figure 1:**
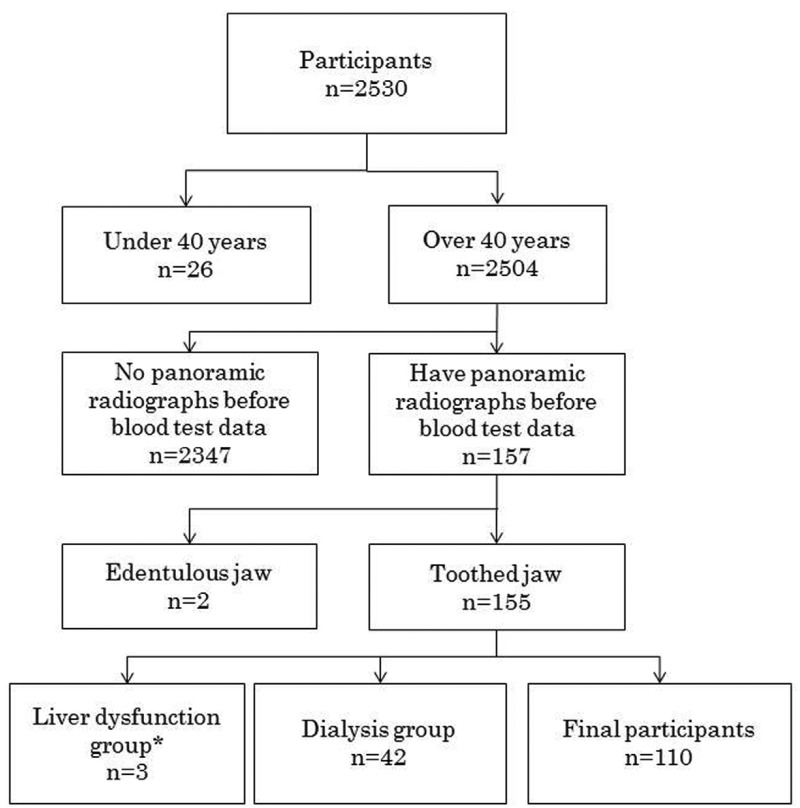
Participant’s flowchart. Exclusion criteria were under 40 years old, absent a panoramic radiograph before the blood test, edentulous jaw, dialysis patients and have liver disfunction. * The people who are diagnosed of “hepatitis B, hepatitis C, and hepatoma” were defined as “Liver dysfunction group”.

The Project in Sado for Total Health (PROST) is executed in Sado Island, Niigata Prefecture Japan. The population was nearly 57,000 as of October 2015. The PROST is a hospital-based cohort study of outpatients of Sado General Hospital that began in June 2008. In the current study, 2,530 individuals between 21 and 102 years of age joined the PROST as of May 2015. All participants underwent a general physical examination, blood test, and interviews for health check from 2008 to 2015. The participants visited the department of dentistry in the hospital and underwent panoramic radiography for dental treatments from 2003 to 2015. The average interval between panoramic radiographs and blood tests was 866 days. History of illness was corrected from clinical record. The study inclusion and exclusion criteria are shown in Figure 1. Systematic disease was not considered expect of liver dysfunction. The people who are diagnosed of “hepatitis B, hepatitis C, hepatoma” were defined as “Liver dysfunction group” A total of 110 participants were finally included in the study. Informed consent was obtained from each participant. We have obtained consent to publish from the participant to report individual patient data. The medical ethics committee of Niigata University approved the study protocol (approval number 511).

-Radiographic analysis

ABL was measured using panoramic radiography (19). We calculated the distance from the cement-enamel junction (CEJ) to the alveolar crest (AC) and the total root length (CEJ-apex) of each remaining tooth at two sites (mesial and distal) using Image J software (Rasband, W.S., ImageJ, U. S. National Institutes of Health, Bethesda, Maryland, USA). The apex was defined as the most apically located point of the tooth. In teeth restored with fillings or crowns, the most apical limit of the restoration was considered equivalent to the CEJ and taken as the reference point. ABL was measured as the percentage of the distance between the CEJ-AC and the CEJ-apex (20). The measurements were made for all remaining teeth including the third molars. Residual roots without a cap for overdenture were excluded. Tooth with caries or periapical lesion was not excluded. Data were collected by four dentists who were calibrated prior to the study using 10 panoramic radiographs (Cronbach alpha coefficient = 0.73). We divided the participants into quartiles according to individual average ABL values.

-Blood test data

Serum concentrations of ALT, aspartate aminotransferase (AST), and gamma-glutamyltransferase (GGT) were measured. The standard values for each serum liver enzymes were based on Japanese Committee for Clinical Laboratory Standard guidelines. Elevated levels were defined as >30 U/L for AST, >42 U/L in men and >23 U/L in women for ALT and >64 U/L in men and >32 U/L in women for GGT.

-Covariates

Alcohol consumption, smoking habits, age, blood pressure, and body mass index (BMI) were considered as covariates, according to previous studies reporting the risk factors for elevated serum liver enzyme levels (16,21-23). Alcohol drinking and smoking habits were surveyed using a self-administered questionnaire. The responses for alcohol drinking habits questionnaire were as follows: (1) “I drink more than 1 day a week, “(2) “I was a drinker before,” (3)” I’m a social drinker,” and (4) ” I do not drink alcohol at all.” This study categorized social drinkers and past drinkers into the “No drinking habits” and “Drinking habits” groups, respectively. Similarly, the responses for smoking habits were as follows: (1) “I have a smoking habit,” (2)” I had a smoking habit before,” and (3) “I do not smoke at all.” Past smokers were categorized into the “Have smoking habits” group.

Age, blood pressure, and BMI were recorded during PROST registration. Blood pressure was measured twice, with average blood pressure ≥140 and/or ≥90 mmHg, and/or the use of antihypertensive medication defined as hypertension.

-Statistical analysis

The participants characteristics (sex, age, number of teeth, systolic blood pressure, diastolic blood pressure, BMI, drinking habit, smoking habit) according to ABL quartiles were evaluated using Mann-Whitney U and chi-square tests. Subsequently, we evaluated the univariate association between liver enzyme levels and the highest ABL quartile or other variables. Then, with GGT, ALT, or AST as the response variables, the associations with the highest quartile of ABL were assessed using multiple logistic regression analysis. The confounding factors were selected based on the results of the univariate analyses. Statistical analyses were performed using SPSS Statistics for Windows, version 23.0 (IBM Corp., Armonk, New York, USA).

## Results

**Figure 2:**
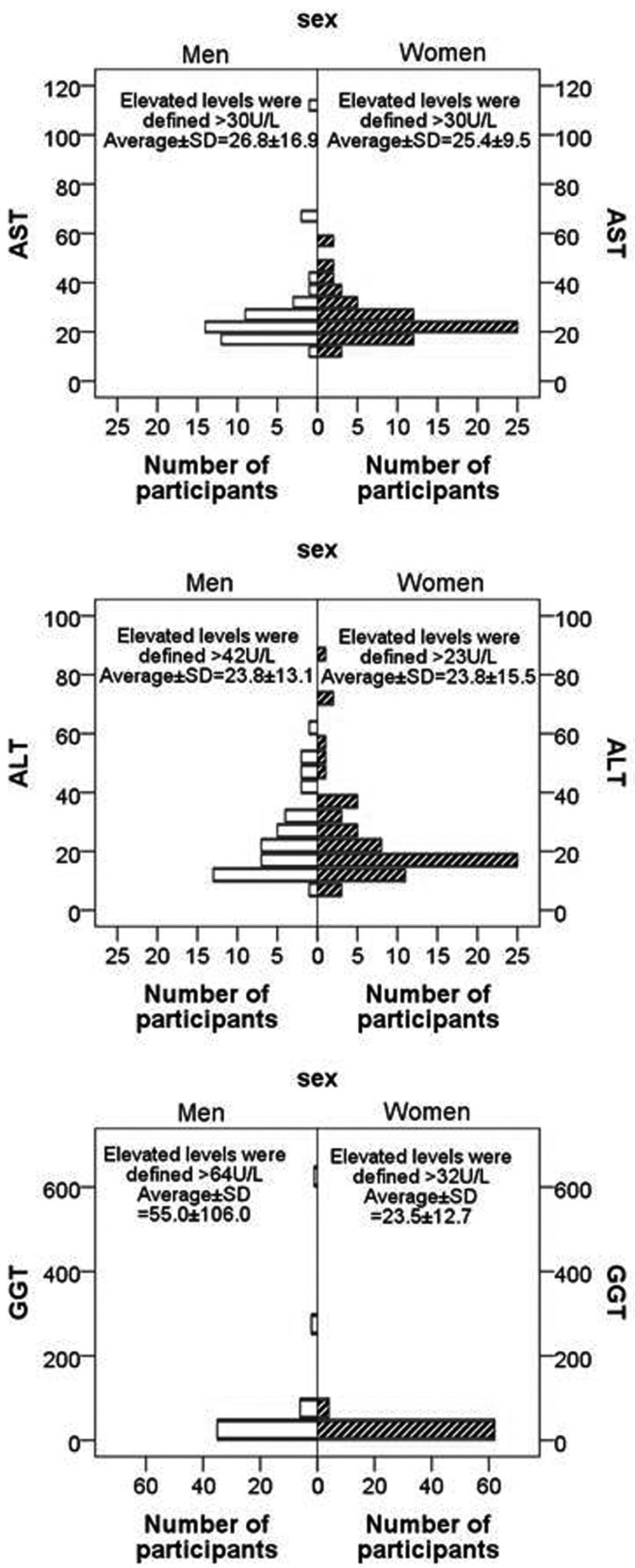
Histograms of serum liver enzymes by gender.

As figure 2, the liver enzymes values were not normally distributed (Kolmogorov-Smirnov test, *p*>0.05). Therefore we determined non-parametric test used.

The study participant characteristics are shown in  Table 1. Significant differences were observed between ABL quartiles in sex, number of teeth and smoking habit.

**Table 1:**
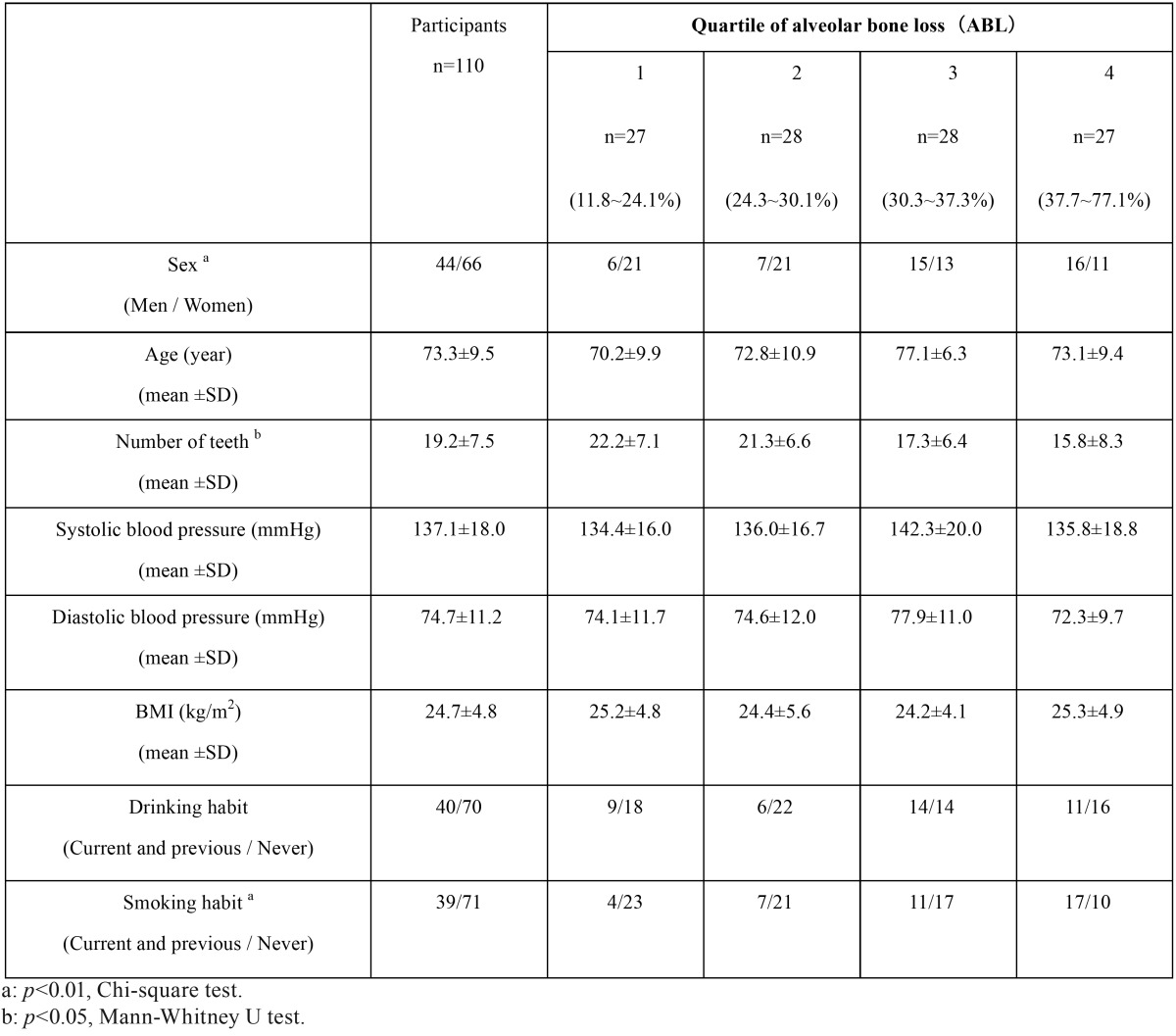
Characteristics of participants.

 Table 2 shows univariate associations of serum liver enzymes with the highest quartile of ABL or other risk factors. We selected the top three variables with the smallest *p*-values as the confounding factors for the subsequent multiple logistic regression analysis. As shown in  Table 3, no significant association was observed between the highest quartile of ABL and elevation of serum liver enzyme levels after adjusting for confounding factors.

**Table 2:**
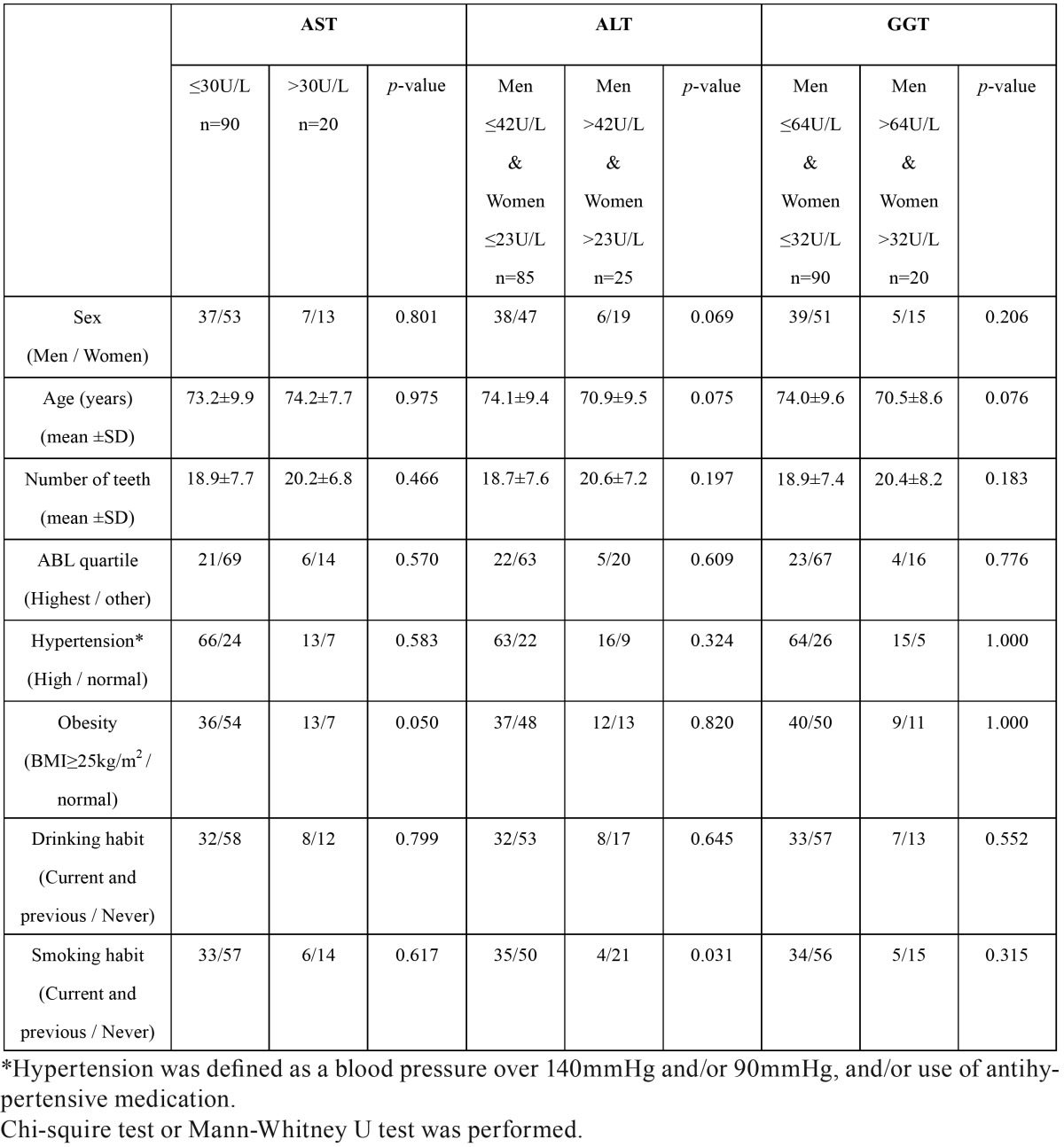
Univariate associations of serum liver enzymes with the highest quartile of ABL or other risk factors.

**Table 3:**
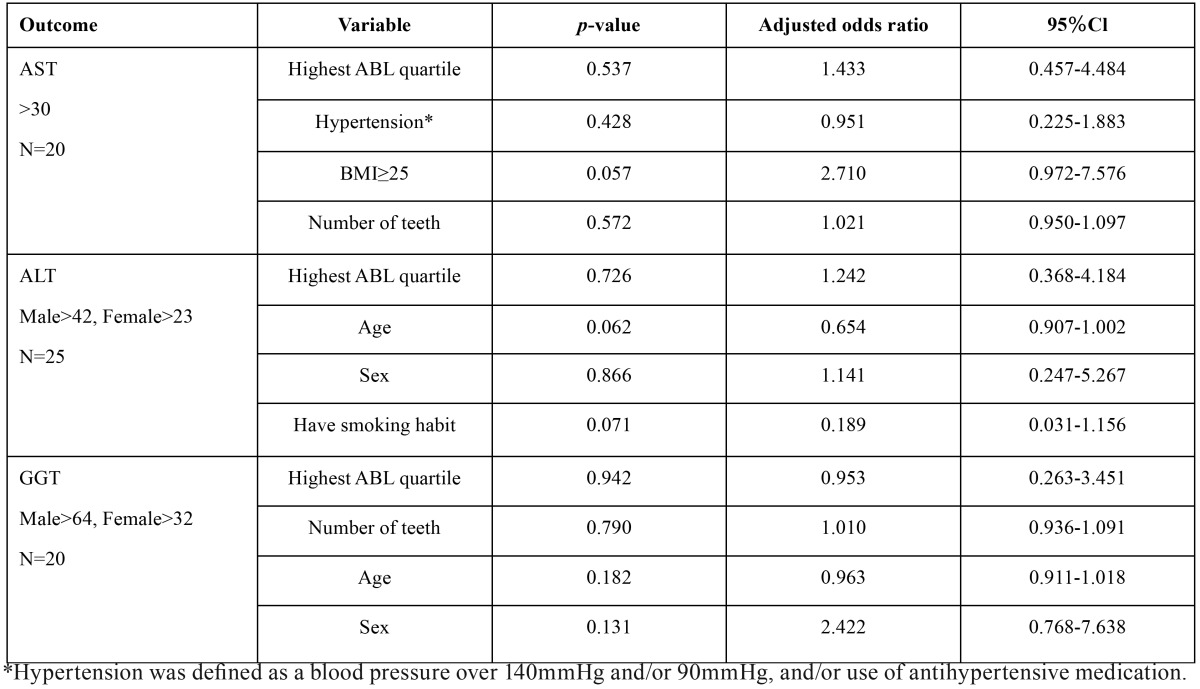
Multiple logistic regression analyses for elevated serum liver enzymes as outcomes.

## Discussion

In this study, we did not find significant association between ABL and serum levels of the AST, ALT, and GGT liver enzymes in Japanese adults, inconsistent with previous studies. The discrepancy might be caused by differences in selected parameters of periodontal diseases. Several parameters such as probing depth, clinical attachment level, or percentage of bleeding on probing, have been used in other studies to assess the possible relevance of periodontal diseases to systemic diseases. Morita *et al.* reported the association between periodontal pocket depth ≥4mm and serum GGT levels (16). Among the parameters, ABL and clinical attachment level represent the result of tissue destruction by periodontitis, which existed in the past (24). They are essential features of periodontitis; in contrast, pocket depth and bleeding on probing are observed even in gingivitis. Bone-resorbing cytokines such as IL-1β, TNF-α, IL-6, and RANKL are produced in reaction to bacterial infections and induce ABL (25-27). Since the degree of ABL could reflect integrated history of inflammation, it was expected to be associated with hepatic dysfunction. The results of this study suggest that inflammatory burden by periodontitis would be a relatively short-term modulator of hepatic function.

Unlike most previous reports on the relationships between periodontal diseases and diagnosed hepatic diseases (15,28,29), we categorized the participant groups using the standard values of serum liver enzymes. These markers can be used as a first step in diagnosis of liver dysfunction in large-sample surveys. Although some previous studies used serum liver enzyme levels, the participants were younger than those in this study (16-18). Additionally, they used data from health checkups, whereas the present study was hospital-based. Furthermore, we found no significant associations between the elevation of serum liver enzymes and known risk factors such as, drinking or smoking habits, sex, obesity, or hypertension after adjusting for other variables. These facts suggest that higher age and some potential confounders may have concealed relationships between ABL and the elevation of serum liver enzymes in this study.

The distributions of the degrees of ABL were skewed according to sex, number of teeth, and smoking habit, in agreement with the findings of previous reports. Female sex, a larger number of teeth, and non-smoking status were associated with lower ABL.

We did not collect bacteriological data such as plaque index or the presence of periodontopathic bacteria which could have been significant confounders. Another limitation of this study was the number of participants. Additionally, we could not test the causative relationships between ABL and the elevation of serum liver enzymes, although the chronological orders were consistent. Longitudinal studies are needed to improve the understanding of the association between elevated serum liver enzymes and periodontal diseases.
